# Autoimmunenzephalitis – ein Update

**DOI:** 10.1007/s00115-022-01411-1

**Published:** 2022-12-14

**Authors:** Josephine Heine, Ankelien Duchow, Rebekka Rust, Friedemann Paul, Harald Prüß, Carsten Finke

**Affiliations:** 1 Klinik für Neurologie, Charité – Universitätsmedizin Berlin, Berlin, Deutschland; 2 Neurocure Clinical Research Center, Charité – Universitätsmedizin Berlin, Corporate Member of Freie Universität Berlin, Humboldt-Universität zu Berlin, & Berlin Institute of Health, 10117 Berlin, Deutschland; 3 Experimental & Clinical Research Center, Charité – Universitätsmedizin Berlin, Corporate Member of Freie Universität Berlin, Humboldt-Universität zu Berlin, & Berlin Institute of Health & Max Delbrück Center for Molecular Medicine, 13125 Berlin, Deutschland; 4 Deutsches Zentrum für Neurodegenerative Erkrankungen (DZNE), Berlin, Deutschland; 5 Berlin School of Mind and Brain, Humboldt-Universität zu Berlin, Berlin, Deutschland

**Keywords:** Autoimmunenzephalitis, NMDA, NMDA-Rezeptor, Enzephalitis, Antikörper, Diagnosekriterien, MRT, Liquor, Therapie, Outcome, Autoimmune encephalitis, NMDA, NMDA receptor, Encephalitis, Antibodies, Diagnostic criteria, MRI, Cerebrospinal fluid, Therapy, Outcomes

## Abstract

Der Nachweis von Autoantikörpern gegen Nerven- oder Gliazellen ermöglicht heute bei zahlreichen neurologischen und psychiatrischen Symptomkomplexen die frühe und spezifische Diagnose einer Autoimmunenzephalitis. Damit hat sich auch die Herangehensweise an die immuntherapeutische Behandlung dieser Krankheitsgruppe fundamental verändert, ebenso wie das Verständnis der zugrunde liegenden Pathophysiologie und der auslösenden Faktoren. Die noch immer wachsende Zahl neuer Autoantikörper erfordert ein regelmäßiges Update über den Stand der Antikörperdiagnostik, die Häufigkeit assoziierter Tumoren sowie das antikörperspezifische Spektrum klinischer Symptome, die von Wesensänderungen und kognitiven Störungen über epileptische Anfälle und Bewegungsstörungen bis hin zu vegetativen und Bewusstseinsstörungen führen. Der Beitrag fasst die aktuellen Neuerungen zusammen, die sich im klinischen Spektrum von Enzephalitiden, in der bildgebenden und Liquordiagnostik, in der Prognoseabschätzung, in der Etablierung innovativer Immuntherapien, in der Anwendung diagnostischer Pfade bereits vor dem Eintreffen des Antikörperbefundes und im Verständnis der Krankheitsentstehung ergeben.

## Abstract (en)

Detection of autoantibodies against neurons and glia cells has brought about the early and specific diagnosis of autoimmune encephalitis in patients with variable neurological and psychiatric symptoms. Growing knowledge not only resulted in profound changes in treatment algorithms including immunotherapy but also in the understanding of disease mechanisms and etiological factors. The still increasing numbers of new autoantibodies calls for continuous updates on the state of the art in antibody diagnostics, frequencies of associated tumors and the clinical spectrum linked to each antibody, which can range from mood changes, cognitive impairment and epileptic seizures to abnormal movements, autonomic dysfunction and impaired levels of consciousness. This article summarizes the recent developments in the predominant clinical presentations of autoimmune encephalitis patients in imaging and cerebrospinal fluid diagnostics and also in prognostic markers, in the establishment of innovative immunotherapies, in the use of diagnostic pathways even before the results of the antibody tests are available and the understanding of the autoimmune etiology.

Die Entdeckung von Autoantikörpern gegen Nerven- oder Gliazellen als Ursache einer autoimmunen Enzephalitis hat in den letzten Jahren zu einer umfassenden Änderung der Herangehensweise an neurologische und psychiatrische Erkrankungen geführt [46]. Dies betrifft nicht nur diagnostische und therapeutische Algorithmen, Aussagen über die Prognose oder die Tumorassoziation, sondern hat auch neue Erkenntnisse zur grundsätzlichen Entstehung von Hirnkrankheiten ergeben und nicht zuletzt die Behandlung etlicher Patienten ermöglicht, deren Symptome zuvor als dissoziativ, infektassoziiert, kryptogen oder „unklar“ eingeschätzt wurden [12, 26]. Parallel zu den wachsenden klinischen Erkenntnissen wurde die Diagnostik deutlich vereinfacht, und sensitive Antikörpertests sind mittlerweile weit verbreitet. Dadurch kann inzwischen bei vielen Patienten eine deutlich beschleunigte Therapieeinleitung erfolgen, die neben der ausreichend wirksamen Therapie als wichtiger Faktor für die langfristige Prognose gilt [30, 58]. Behandlungsansätze richten sich mittlerweile auf verschiedene Zellen und Rezeptoren des Immunsystems und die Zahl der Therapieoptionen wächst kontinuierlich. Zum Teil stecken hinter einzelnen Antikörpern sehr spezifische klinische Syndrome, die epileptische Anfälle, Bewusstseinsstörungen, Bewegungsstörungen, kognitive Störungen, vegetative Störungen oder Psychosen umfassen können. Ein Ende dieser Entwicklung ist derzeit noch nicht absehbar, da weiterhin neue Autoantikörper entdeckt werden, deren pathogenetische Bedeutung in zukünftigen Studien untersucht werden wird. Dieser Beitrag beschreibt die derzeit wichtigsten Autoantikörper, die als Ursache einer autoimmunen Enzephalitis auftreten können, umreißt gängige diagnostische und therapeutische Prinzipien und benennt neue Erkenntnisse, die für das Verständnis der Krankheitsentstehung von Enzephalitiden relevant sind.

## Klinische Präsentation

Autoimmunenzephalitiden können in Varianten mit Antikörpern gegen intrazelluläre Antigene und Varianten mit Antikörpern gegen Oberflächenantigene unterschieden werden.

### Enzephalitis mit Antikörpern gegen intrazelluläre Antigene

Sehr häufig liegt der Autoimmunenzephalitis mit Antikörpern gegen intrazelluläre Zielstrukturen ein Tumor zugrunde. Die neuronalen Antigene werden z. B. bei Thymomen, kleinzelligen Lungenkarzinomen, Ovarial- oder Hodenkarzinomen ektop exprimiert und führen zur Bildung sog. onkoneuraler Antikörper. Diese nicht direkt pathogenen Antikörper gegen intrazelluläre Zielstrukturen sind mit einer zytotoxischen T‑Zell-Antwort assoziiert, die für die neuronale Schädigung verantwortlich ist. Da Enzephalitiden mit Antikörpern gegen intrazelluläre Antigene nur schlecht auf eine Immuntherapie ansprechen, ist eine frühzeitige Tumorsuche entscheidend für Therapieerfolg und Prognose. Der Nachweis bestimmter Antikörpertypen kann hierbei die Suche nach dem Primärtumor deutlich vereinfachen (Tab. 1).

**Table 1:** 

Antikörper	Assoziierte klinische Syndrome	Tumorassoziation
Amphiphysin	Limbische Enzephalitis, Enzephalomyelitis, sensible Neuropathie, Stiff-Person-Syndrom, zerebelläre Degeneration	> 90 %, meist Mammakarzinom oder kleinzelliges Bronchialkarzinom (SCLC)
ANNA‑3	Limbische Enzephalitis, Kleinhirndegeneration, Enzephalomyelitis, sensible Neuropathie	≈ 60 % SCLC
CV2 (CRMP-5)	Limbische Enzephalitis, Bewegungsstörungen, Opsoklonus, Optikusneuritis, Enzephalomyelitis, zerebelläre Degeneration, NMOSD	> 90 %, meist SCLC oder Thymome
GAD^a^	Stiff-Person-Syndrom, zerebelläre Ataxie, limbische Enzephalitis, therapierefraktäre epileptische Anfälle Seltener paraneoplastisch, dann meist verbunden mit Opsoklonus-Myoklonus-Syndrom und Enzephalomyelitis Häufig assoziiert mit weiteren Autoimmunerkrankungen (z. B. Typ 1-Diabetes)	Selten; Thymom, neuroendokriner Tumor oder Lungen‑/Mammakarzinom möglich
GFAP	Meningoenzephalitis, Meningoenzephalomyelitis (in 10–20 % zusätzlich NMDAR-Ak nachweisbar)	≈ 10–20 %, meist Ovarialteratom wenn NMDAR-Ak nachweisbar (sonst mit diversen Tumorentitäten assoziiert)
Hu (ANNA-1)	Limbische Enzephalitis, Hirnstammenzephalitis, zerebelläre Degeneration, Denny-Brown-Syndrom, Dysautonomie, Myelitis, seltener motorische Neuropathie, vereinzelt Chorea, LEMS, Opsoklonus-Myoklonus-Syndrom (bei pädiatrischem Neuroblastom)	> 90 %, meist SCLC (besonders bei LEMS), auch bei Neuroblastom oder Prostatakarzinom
Ma2	Limbische Enzephalitis, zerebelläres Syndrom, Ataxie	> 90 %, meist testikulärer oder Lungentumor
Ri (ANNA-2)	Hirnstammenzephalitis, Opsoklonus-Myoklonus-Syndrom, zerebelläre Ataxie	> 90 %, meist Mammakarzinom, gynäkologische Tumoren oder SCLC
Sox‑1 (AGNA)	Lambert-Eaton-Myasthenie-Syndrom (LEMS), zerebelläre Ataxie, Polyneuropathie	> 90 %, meist SCLC (v. a. bei LEMS)
Yo (PCA-1)	Zerebelläres Syndrom mit Ataxie, Dysarthrie, Nystagmus; mögliche Begleitsymptome: Diplopie, Dysphagie, periphere Neuropathie	> 90 %, meist Mammakarzinom oder andere gynäkologische Tumoren, SCLC
Zic4	Zerebelläre Degeneration, Ataxie	> 90 %, meist SCLC

*LEMS* Lambert-Eaton-Myasthenie-Syndrom, *SCLC* kleinzelliges Bronchialkarzinom („small-cell lung cancer“), *NMOSD* Neuromyelitis optica spectrum disorder

^a^Autoantikörper gegen Glutamat-Decarboxylase (*GAD*) bilden eine gewisse Ausnahme. Zwar richten sich diese gegen ein intrazelluläres Antigen, jedoch ist die GAD-Enzephalitis in den meisten Fällen nicht mit einem Tumor assoziiert. Die GAD-Enzephalitis hat stattdessen häufig eine chronische Verlaufsform und spricht meist nur eingeschränkt auf eine Immuntherapie an

Eine gewisse Ausnahme spielen Autoantikörper gegen Glutamat-Decarboxylase (GAD), da diese zwar gegen ein intrazellulär lokalisiertes Antigen gerichtet, in den meisten Fällen aber nicht mit einem Tumor assoziiert sind [2]. Hohe Titer (> 10.000 IU/ml) sind bei einer GAD-Enzephalitis mit limbischer Enzephalitis, Stiff-Person-Syndrom, zerebellärer Ataxie oder therapierefraktären epileptischen Anfällen assoziiert [42]. Die GAD-Enzephalitis hat häufig eine chronische Verlaufsform mit unvollständiger Genesung und spricht nur eingeschränkt auf eine Immuntherapie an [57]. Im Gegensatz dazu gehen niedrigere Titer mit einem heterogenen Symptomspektrum einher, bei denen in Abwesenheit der typischen Klinik Differenzialdiagnosen erwogen werden sollten.

### Enzephalitis mit Antikörpern gegen neuronale Oberflächenantigene

In den letzten Jahren wurden zahlreiche weitere Antikörper identifiziert, die sich gegen oberflächlich gelegene neuronale Strukturen richten. Diese Oberflächenantigene, wie z. B. NMDA- oder AMPA-Rezeptoren, sind den Antikörpern direkt zugänglich. Oberflächenantikörper wirken daher – im Gegensatz zu intrazellulären Antikörpern – direkt pathogen [11, 13, 46]. Diese Form der Autoimmunenzephalitis spricht besser auf eine Immuntherapie an und ist seltener mit Tumoren assoziiert als klassische paraneoplastische Enzephalitiden mit Antikörpern gegen intrazelluläre Antigene. Einige der häufigsten Krankheitsbilder werden im Folgenden näher beschrieben (Tab. 2).

**Table 2:** 

Enzephalitiden mit Antikörpern gegen Oberflächenantigene				
Neuronale Oberflächenantikörper gegen:	Symptomatik/assoziiertes Syndrom	MRT-Befunde	Mögliche Tumorassoziation	Erkrankungsalter und Geschlecht
NMDA-Rezeptor [12]	Unspezifisches Prodrom (≈ 70 % mit Kopfschmerzen oder Fieber), gefolgt von Verhaltensveränderungen, Psychose, affektiven Störungen, Gedächtnisstörungen, epileptische Anfälle, Bewegungsstörungen wie orofaziale Dyskinesien, autonome Instabilität, Bewusstseinsstörungen	Meist unauffällig; kleinere, unspezifische Läsionen der weißen Substanz im T2/FLAIR-gewichteten MRT in 25–50 % der Fälle, seltener Veränderungen der Basalganglien, des Hirnstamms und Zerebellums, im PET frontotemporaler Hypermetabolismus und okzipitaler Hypometabolismus, im Follow-up Hippokampusatrophie möglich	≈ 40 % in jüngerem Erkrankungsalter (12–45 Jahre, meist Ovarialteratome), ≈ 25 % bei älterem Erkrankungsalter (ab 45 Jahre, vorrangig Karzinome)	w > m (3:1) Jeden Alters, häufig Kindheit oder frühes Erwachsenenalter
LGI1 [36, 38]	In der Frühphase pathognomonische faziobrachiale dystone Anfälle (FBDS), gefolgt von limbischer Enzephalitis mit Gedächtnisdefiziten, Temporallappenanfällen, Schlafstörungen und Hyponatriämie	Während der FBDS zumeist unauffällig, bei limbischer Enzephalitis typischerweise mesiotemporale T2-FLAIR-Signalanhebungen und häufig PET-Hypermetabolismus der Basalganglien und des medialen Temporallappens, im Follow-up ist eine Hippokampusatrophie typisch (häufig auch Hinweis auf Hippokampussklerose)	Selten (< 10 %), meist Thymome	m > w (2:1) > 40 Jahre
CASPR2 [39]	Limbische Enzephalitis, Morvan-Syndrom, Neuromyotonie	Typischerweise unauffälliges MRT bei Morvan-Syndrom, in einigen Fällen frontale und mesiotemporale T2-FLAIR-Signalanhebungen oder unspezifische periventrikuläre Läsionen, seltener Entwicklung einer Hippokampusatrophie, im PET sind auch bei unauffälligem MRT Veränderungen in frontotemporalen Bereichen und den Basalganglien möglich	Selten, Thymom möglich	m > w (9:1) Spätes Erwachsenenalter
AMPA-Rezeptor [33]	Limbische Enzephalitis mit ausgeprägten Gedächtnisstörungen und Konfabulationen, seltener rein psychiatrische Symptomatik Bei ≈ 30 % Nachweis weiterer Antikörper	Typischerweise mesiotemporale Signalanhebungen im T2-FLAIR-MRT in 90 % der Fälle, im Follow-up können sich Hinweise auf eine Hippokampussklerose zeigen	≈ 70 %, meist Lungenkarzinom, Mammakarzinom oder Thymom	w > m (2,3:1) Spätes Erwachsenenalter
GABA-A-Rezeptor [55]	Häufig therapieresistente epileptische Anfälle und Status epilepticus, kognitive Defizite, Desorientierung, Gedächtnisstörungen, Depression, Psychose, Mutismus	Ausgeprägte multifokale oder diffuse kortikale und subkortikale T2-FLAIR-Signalanhebungen, schnelle Progression zu Atrophie und bilateralen Läsionen in einigen Fällen	Selten, Hodgkin-Lymphom möglich	m > w (1,5:1) Frühes Erwachsenenalter
GABA-B-Rezeptor [40]	Limbische Enzephalitis mit häufigen epileptischen Anfällen, seltener zerebelläre Ataxie oder Hirnstammbeteiligung	Typischerweise mesiotemporale T2-FLAIR-Signalanhebungen, in einzelnen Fällen verbunden mit frontotemporaler Leukenzephalopathie oder extensiveren Läsionen (einschließlich Hirnstamm, Zerebellum und Basalganglien), im Follow-up können sich eine frontotemporale und Hippokampusatrophie entwickeln	≈ 70 %, meist kleinzelliges Bronchialkarzinom oder neuroendokrine Tumoren	m = w Spätes Erwachsenenalter
mGluR5 [56]	Limbische Enzephalitis, Ophelia-Syndrom Verhaltensveränderungen, Gedächtnisdefizite, Depression, Wahnvorstellungen	Auffälliges MRT bei ≈ 50 % mit T2-FLAIR-Signalanhebungen mit in limbischen und extralimbischen Arealen	Häufig, meist Hodgkin-Lymphom	m > w (1,5:1) Frühes Erwachsenenalter
DPPX [29]	Limbische Enzephalitis, Tremor, Myoklonus, Halluzinationen, Desorientierung, Agitation, epileptische Anfälle, therapierefraktäre Diarrhö	Typischerweise unauffälliges MRT, in einigen Fällen unspezifische T2-FLAIR-Signalanhebungen in der periventrikulären oder subkortikalen weißen Substanz	Keine bekannte Tumorassoziation	m > w (2,3:1) Spätes Erwachsenenalter
Glycin-Rezeptor [8, 35]	PERM, Stiff-Person-Syndrom, Dysautonomie, kognitive Defizite, Halluzinationen, Depression, Ängstlichkeit	Häufig unauffällige Bildgebung, in einigen Fällen T2-hyperintense Läsionen der weißen Substanz, spinale Läsionen oder mesiotemporale Signalanhebungen/Atrophie	< 10 %, meist Thymome	m = w Spätes Erwachsenenalter
IgLON5 [24]	Schlafstörungen, Schlafapnoe, Stridor, Dysarthrie, Dysphagie, Dysautonomie, Bewegungsstörungen, kognitive Defizite	Typischerweise unauffälliges MRT	Keine bekannte Tumorassoziation	m = w Spätes Erwachsenenalter

*AMPA* „α-amino-3-hydroxy-5-methyl-4-isoxazolepropionic acid“, *CASPR2* „contactin-associated protein-like 2“, *DPPX* „dipeptidyl-peptidase-like protein 6“, *FBDS* faziobrachiale dystone Anfälle, *GABA* „γ-aminobutyric acid“, *LGI1* „leucine-rich glioma inactivated 1“, *NMDA* N-Methyl-D-Aspartat, *PERM* progressive Enzephalomyelitis mit Rigidität und Myoklonien, *PET* Positronenemissionstomographie

#### NMDA-Rezeptor-Enzephalitis

**Figure 1:**
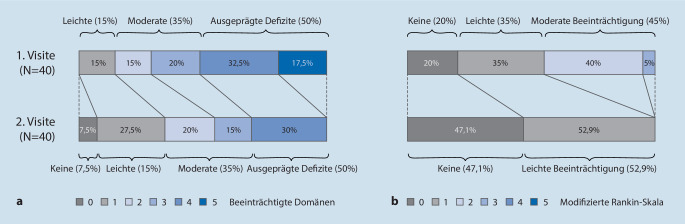

Als eine der häufigsten Formen der Autoimmunenzephalitis ist die Enzephalitis mit Antikörpern gegen den NMDA-Rezeptor (NMDAR) inzwischen sehr gut charakterisiert (für spezifische Diagnosekriterien siehe auch [26]). Ihr charakteristischer Verlauf beginnt in vielen Fällen mit einer grippeähnlichen Prodromalphase und entwickelt sich innerhalb kurzer Zeit zu einem komplexen neuropsychiatrischen Krankheitsbild, beginnend zunächst häufig mit psychiatrischen Symptomen wie Verhaltensveränderungen, Wahnvorstellungen oder affektiven Symptomen [12]. Im weiteren Verlauf treten fast immer neurologische Symptome hinzu, u. a. epileptische Anfälle, Bewegungsstörungen, autonome Dysregulation und Bewusstseinsstörungen, die bei einigen Patienten eine intensivmedizinische Behandlung erfordern [50]. Bis zu 40 % der betroffenen Patienten sind Kinder, die etwas häufiger als Erwachsene an Bewegungsstörungen und Sprachstörungen leiden [59]. Nahezu alle Patienten zeigen zudem ausgeprägte Gedächtnisstörungen [20, 30]. Diese können, zusammen mit Beeinträchtigungen der Exekutivfunktionen, auch noch mehrere Jahre nach der Akutphase fortbestehen – selbst, wenn die neurologische Beeinträchtigung (modifizierte Rankin-Skala) nahezu vollständig zurückgegangen ist (Abb. 1; [30]).

**Figure 2:**
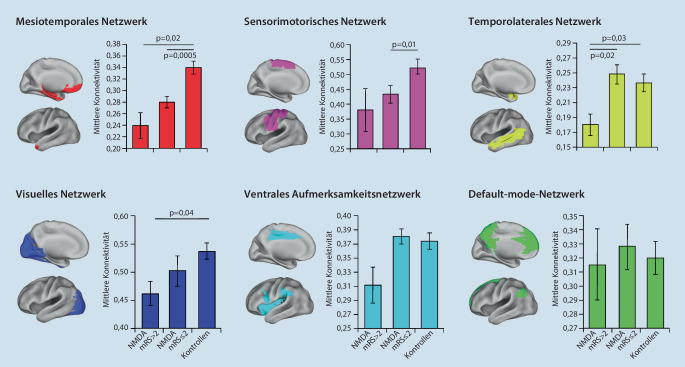

Im Liquor ist meist eine geringfügige Pleozytose nachweisbar, oligoklonale Banden können im Verlauf auftreten [14]. MRT-Auffälligkeiten sind in der Regel unspezifisch, korrelieren nicht mit der klinischen Symptomatik (unspezifische kleine Läsionen der weißen Substanz in frontalen, parietalen und mesiotemporalen Arealen; gelegentlich Thalamus, Kleinhirn und Hirnstamm, seltener Basalganglien) und sind in der Akutphase nur bei ca. 25–50 % der Patienten nachweisbar. Quantitative Analysemethoden können jedoch im Langzeitverlauf eine Volumenminderung des Hippocampus [19] und Veränderungen in der weißen Substanz mittels Diffusionsbildgebung nachweisen [21]. Die Mehrzahl der Kinder mit NMDAR-Enzephalitis weist eine relevante Hirnvolumenminderung und eine Beeinträchtigung des altersgerechten Hirnwachstums auf [5]. Mittels funktioneller MRT (sog. „Resting-State-MRT“) konnte kürzlich gezeigt werden, dass auch bei weitgehend unauffälliger klinischer Bildgebung die intrinsische Netzwerkorganisation noch Jahre nach einer NMDAR-Enzephalitis beeinträchtigt sein kann (Abb. 2; [19, 44, 51]).

Neben Ovarialteratomen wurden auch Virusinfektionen des Gehirns als Triggerfaktor einer Autoimmunenzephalitis identifiziert. Besonders klar ist dieser Zusammenhang für Enzephalitiden mit Herpes-simplex-Virus Typ 1, die in fast 30 % der Fälle zu einer sekundären NMDA-Rezeptor-Enzephalitis führen [3, 47]. Mittlerweile sind auch zahlreiche andere Viruserkrankungen im Zusammenhang mit einer NMDA-Rezeptor-Enzephalitis beschrieben worden, was für einen breiteren pathophysiologischen Zusammenhang spricht [45]. Interessanterweise wurden sogar im Rahmen der aktuellen COVID-19-Pandemie Patienten mit NMDA-Rezeptor-Enzephalitis nach Infektion mit SARS-CoV‑2 identifiziert, [43] und schwerkranke COVID-19-Patienten mit neurologischen Symptomen zeigten gehäuft multiple ZNS-Autoantikörper im Liquor [23]. Aktuelle Forschungen beschäftigen sich mit der Frage, ob einige virusneutralisierende Antikörper gleichzeitig körpereigene Antigene erkennen und somit für neurologischen Beschwerden mitverantwortlich sein können [37]. Sollte sich dieser Verdacht wissenschaftlich erhärten lassen, dürften zahlreiche neue Erkenntnisse zur Interaktion des Nervensystems und Immunsystems zu erwarten sein, die die Herangehensweise an neurologische und psychiatrische Symptome grundsätzlich verändern könnten.

#### LGI1-Enzephalitis

Faziobrachiale dystone Anfälle (FBDS) sind pathognomonisch für das Vorliegen von LGI1-Antikörpern [41]. Es handelt sich dabei um kurze dystone Anspannungen von Gesicht und Arm einer Körperhälfte, die mit hoher Frequenz auftreten können (100 FBDS/Tag). FBDS sprechen sehr gut auf eine Immuntherapie an, jedoch schlecht auf antikonvulsive Medikationen [58]. Sie sind zudem Vorboten einer Progression der Erkrankung zum Stadium der limbischen Enzephalitis mit Gedächtnisstörungen und Temporallappenanfällen – diese Progression kann jedoch durch eine frühzeitige Immuntherapie verhindert werden. Diagnostisch fällt bei der LGI1-Enzephalitis zudem häufig eine Hyponatriämie auf. Liquoruntersuchungen sind in der Regel unauffällig.

**Figure 3:**
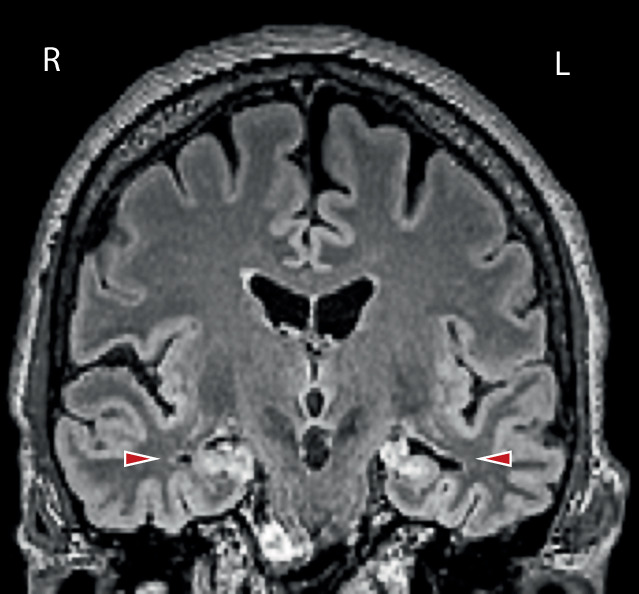

Die MRT-Bildgebung im Verlauf reicht vom zu Beginn unauffälligen Befund über ein typisch limbisches Befallsmuster bis hin zu hippokampaler Atrophie (Abb. 3; [1, 53, 54]). Kurz- und Langzeitgedächtnisdefizite können auch noch Jahre nach einer LGI1-Enzephalitis bestehen bleiben [22]. Neben einem selektiven Volumenverlust im Hippokampus finden sich auch Veränderungen in der Konnektivität funktioneller Netzwerke [32]. Obwohl initial vor allem der mediale Temporallappen betroffen ist, können durch pathologische Langzeitveränderungen der intrinsischen Netzwerkorganisation im fMRT auch andere Hirnbereiche funktionell betroffen sein.

#### CASPR2-Enzephalitis

Das kaliumkanalkomplexassoziierte Protein CASPR2 („contactin-associated protein 2“) wird im Hippokampus, im Kleinhirn und an peripheren Nerven exprimiert. CASPR2-Ak können daher sowohl mit einer Übererregbarkeit peripherer Nerven (u. a. mit Neuromyotonie, neuropathische Schmerzen) als auch mit einer limbischen Enzephalitis assoziiert sein. Beim Morvan-Syndrom besteht eine Kombination aus neuropsychiatrischen Symptomen (mit häufig ausgeprägter Schlafstörung und kognitiven Defiziten), autonomer Funktionsstörung und Neuromyotonie. Betroffen sind überwiegend ältere Männer um die 6. Lebensdekade. Im MRT sind bei limbischer Enzephalitis häufig uni- oder bilaterale mediotemporale T2/FLAIR-Signalintensitätssteigerungen nachweisbar; im Falle eines Morvan-Syndroms findet sich in der Mehrzahl ein unauffälliger Befund [31, 39]. Im Liquor sind bei ca. einem Viertel der Patienten eine Pleozytose oder oligoklonale Banden nachweisbar [7].

#### AMPA-Rezeptor-Enzephalitis

Bei Patienten mit AMPAR(Anti-α-Amino-3-Hydroxy-5-Methyl-4-Isoxazolpropionsäure-Rezeptor)-Enzephalitis entwickelt sich eine akute limbische Enzephalitis mit epileptischen Anfällen, Gedächtnisstörungen und Psychose; auch eine Präsentation mit isolierter Psychose wurde berichtet [33]. In einigen Fällen treten Schlaf- und Bewegungsstörungen auf. Die AMPAR-Enzephalitis ist in etwa 70 % der Fälle paraneoplastisch. Das MRT zeigt T2-FLAIR-Signalanhebungen, insbesondere im medialen Temporallappen, sowie subkortikale und kortikale Läsionen, die auf eine Demyelinisierung hindeuten können [31]. Die Untersuchung des Liquors weist in etwa der Hälfte der Fälle eine Pleozytose auf [33].

#### GABA_A_-Rezeptor-Enzephalitis

Die Enzephalitis mit Antikörpern gegen den inhibitorischen GABA_A_-Rezeptor des Chloridionenkanals manifestiert sich mit epileptischen Anfällen, v. a. Epilepsia partialis continua und therapierefraktärem Status epilepticus. Darüber hinaus können psychiatrische Symptome (Halluzinationen, Verhaltensänderungen) und kognitive Defizite bestehen [55]. Motorische Auffälligkeiten, vor allem bei Kindern, betreffen häufig die Gesichtsmuskulatur (orofaziale Dyskinesien, Krämpfe). Das MRT weist bei fast allen Patienten sehr prominente Veränderungen auf, insbesondere multifokale kortikale und subkortikale T2/FLAIR-hyperintense Läsionen v. a. im Temporal- und Frontallappen [31]. Im Liquor findet sich bei ≈ 60 % der Patienten eine Pleozytose und oligoklonale Banden [55].

#### GABA_B_-Rezeptor-Enzephalitis

In etwa der Hälfte der Fälle liegen dem Krankheitsbild mit typischer limbischer Enzephalitis und häufigen epileptischen Anfällen ein kleinzelliges Bronchialkarzinom oder neuroendokrine Tumoren zugrunde [40]. Entsprechend fallen im MRT ein- oder beidseitige T2/FLAIR-Signalintensitäten des medialen Temporallappen auf, teilweise mit frontotemporaler oder hippokampaler Atrophie [31].

#### mGluR5-Enzephalitis

Antikörper gegen den metabotropen Glutamat-Rezeptor 5 (mGluR5) sind mit komplexen neuropsychiatrischen Syndromen assoziiert, inkl. Depressivität, Psychose mit Halluzinationen, Persönlichkeits- und Verhaltensstörungen und emotionaler Instabilität. Zusätzlich können epileptische Anfälle, Bewegungsstörungen, Bewusstseinsstörungen und Schlafstörungen bestehen. Bei ca. 50 % der Patienten kann ein Hodgkin-Lymphom nachgewiesen werden (dann als Ophelia-Syndrom bezeichnet; [56]). Im Liquor finden sich häufig eine Pleozytose und oligoklonale Banden. T2/FLAIR-Veränderungen im MRT treten sowohl in limbischen als auch in extralimbischen Arealen auf.

#### DPPX-Enzephalitis

Eine limbische Enzephalitis in Kombination mit Tremor, Myoklonien, Hyperekplexie und Halluzinationen wird im Zusammenhang mit Antikörpern gegen das „dipeptidyl-peptidase-like protein 6“ (DPPX) beobachtet [29]. Häufig geht eine prodromale Phase mit schweren Diarrhöen und Gewichtsverlust voraus, die sich als therapierefraktär erweisen kann. Das MRT zeigt in der Regel normale oder unspezifische Veränderungen, in einzelnen Fällen sind fleckförmige periventrikuläre und subkortikale T2/FLAIR-Signalintensitäten der weißen Substanz beschrieben [31].

#### Glycin-Rezeptor-Enzephalitis

Antikörper gegen den im Hirnstamm und Rückenmark lokalisierten Glycin-Rezeptor können eine progressive Enzephalomyelitis mit Rigidität und Myoklonien (PERM) oder ein Stiff-Person-Syndrom hervorrufen, begleitet von kognitiven und psychiatrischen Symptomen [8]. Bei bis zu 10 % der Patienten finden sich Thymome. In der Mehrheit der Fälle (ca. 70 %) zeigt das MRT einen unauffälligen Befund, beobachtet werden jedoch auch T2/FLAIR-Hyperintensitäten der weißen Substanz, Signalauffälligkeiten und Atrophie der Temporallappen sowie spinale Läsionen [31].

#### GFAP-Meningoenzephalitis

**Figure 4:**
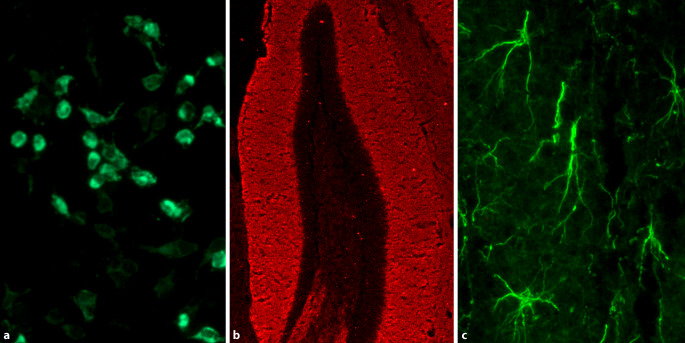

Die GFAP(„glial fibrillary acidic protein“)-Enzephalitis ist eine kürzlich beschriebene Form der autoimmunen Meningoenzephalitis (Abb. 4c; [18]). Die GFAP-Enzephalitis beginnt häufig mit starken Kopfschmerzen und kann sich dann als immuntherapieresponsive Enzephalopathie, Myelitis, Papillitis, zerebelläres Syndrom, autonome Dysfunktion und kognitive Symptome manifestieren [17]. Der Liquor zeigt häufig eine Pleozytose, in etwa der Hälfte der Fälle findet sich im MRT ein charakteristisches radiales Muster einer periventrikulären Kontrastmittelaufnahme.

#### IgLON5-assoziierte Enzephalopathie

Die IgLON5-assoziierte Enzephalopathie steht für einen Paradigmenwechsel in der Neurologie, da die Erkrankung sowohl neuroinflammatorische als auch neurodegenerative Komponenten aufweist: Die Antikörper sind gegen das auf der Zelloberfläche gelegene Adhäsionsprotein IgLON5 gerichtet, andererseits zeigten neuropathologische Untersuchungen aber mehrheitlich Ablagerungen von hyperphosphoryliertem Tau im Hypothalamus, Hippokampus und Hirnstamm. Klinisch manifestiert sich die IgLON5-Enzephalopathie mit Schlafstörungen (Parasomnien mit zielgerichteten Handlungen, periodische nächtliche Beinbewegungen [PLMS]), Atemstörungen (obstruktive Schlafapnoe, Stridor, zentrale Hypoventilation), Hirnstammsymptomen (u. a. Dysarthrie, Dysphagie, Okulomotorikstörungen), autonomer Funktionsstörung und kognitiven Defiziten [28]. Im MRT finden sich keine spezifischen Veränderungen, die Basisparameter des Liquors sind zumeist unauffällig [24]. Im Gegensatz zu der Mehrzahl der Patienten mit Antikörpern gegen Oberflächenantigene sprechen die meisten Patienten mit IgLON5-Tauopathie weniger deutlich auf eine Immuntherapie an [34].

## Diagnostik

Die Diagnostik von Autoimmunenzephalitiden basiert auf Anamnese, klinischer Präsentation, MRT, Liquor, ggf. EEG und Antikörperdiagnostik. Die 2016 veröffentlichten Diagnosekriterien (Tab. 3; [26]) unterstützen die Zuordnung der erhobenen Befunde in den Kategorien einer möglichen Autoimmunenzephalitis, einer definitiven limbischen Enzephalitis und einer trotz negativem Antikörpernachweis wahrscheinlichen Autoimmunenzephalitis.

**Table 3:** 

**Kriterien für eine *mögliche* Autoimmunenzephalitis**	
Alle drei genannten Kriterien müssen erfüllt sein:	
1)	Subakuter Beginn innerhalb von 3 Monaten: Einschränkungen des Kurzzeitgedächtnisses, Bewusstseinsveränderungen oder psychiatrische Symptome
2)	Mindestens einer der folgenden Punkte: – Neue fokale ZNS-Symptomatik – Epileptische Anfälle ohne bekannte Ursache – Liquorpleozytose (Leukozytenzahl > 5/mm^3^) – MRT-Befund suggestiv für Enzephalitis
3)	Ausschluss alternativer Ursachen (infektiologisch, vaskulär, neoplastisch, metabolisch)
**Kriterien für eine *definitive*** ***limbische* Autoimmunenzephalitis**	
Alle vier genannten Kriterien müssen erfüllt sein:	
1)	Subakuter Beginn innerhalb von 3 Monaten: Einschränkungen des Kurzzeitgedächtnisses, epileptische Anfälle oder psychiatrische Symptome, die eine Beteiligung des limbischen Systems vermuten lassen
2)	Bilaterale Auffälligkeiten begrenzt auf die medialen Temporallappen im MRT (T2/FLAIR-Wichtung)
3)	Mindestens einer der folgenden Punkte – Liquorpleozytose (Leukozytenzahl > 5/mm^3^) – EEG: epileptische/slow-wave-Aktivität im Temporallappen
4)	Ausschluss alternativer Ursachen (infektiologisch, vaskulär, neoplastisch, metabolisch)
**Kriterien für eine *antikörpernegative, wahrscheinliche* Autoimmunenzephalitis**	
Alle vier genannten Kriterien müssen erfüllt sein:	
1)	Subakuter Beginn innerhalb von 3 Monaten: Einschränkungen des Kurzzeitgedächtnisses, Bewusstseinsveränderungen oder psychiatrische Symptome
2)	Ausschluss gut beschriebener autoimmuner enzephalitischer Syndrome (z. B. typische limbische Enzephalitis, Bickerstaff-Hirnstammenzephalitis, ADEM)
3)	Abwesenheit gut charakterisierter Autoantikörper in Serum und Liquor sowie mindestens zwei der folgenden Punkte: – MRT-Befund suggestiv für Autoimmunenzephalitis – Liquor: Pleozytose, Nachweis isolierter oligoklonaler Banden und/oder erhöhter IgG-Index – Hirnbiopsie mit inflammatorischen Infiltraten unter Ausschluss anderer Ursachen (z. B. Tumor)
4)	Ausschluss alternativer Ursachen (z. B. infektiologisch, vaskulär, neoplastisch, metabolisch)

*FLAIR* „fluid attenuated inversion recovery“, *ADEM* akute disseminierte Enzephalomyelitis

### MRT

Das zerebrale MRT ist eine essenzielle Standarduntersuchung bei Patienten mit Verdacht auf Autoimmunenzephalitis, insbesondere auch zum Ausschluss möglicher Differenzialdiagnosen. Die MRT-Befunde bei Autoimmunenzephalitiden sind vielfältig. So ist bei der Mehrzahl der Patienten mit NMDAR-Enzephalitis (bis zu 75 %) das MRT unauffällig; auch bei der paraneoplastischen zerebellären Degeneration wird eine Atrophie des Kleinhirns erst im Verlauf nachweisbar [31]. Bei Patienten mit einer limbischen Enzephalitis findet sich hingegen häufig eine uni- oder bilaterale T2/FLAIR-hyperintense Darstellung des medialen Temporallappens (MTL) – die dann (bei bilateraler Affektion) die Diagnose einer „definitiven limbischen Enzephalitis“ erlaubt (Tab. 3; [26]). Bei unilateraler Affektion sollte differenzialdiagnostisch jedoch unter anderem ein Gliom erwogen werden [4]. T2/FLAIR-Hyperintensitäten des MTL lassen sich jedoch nicht bei allen Patienten mit einer LGI1-Ak-assoziierten limbischen Enzephalitis nachweisen, obwohl nahezu alle Patienten im Verlauf eine hippokampale Atrophie entwickeln [22]. Das Ausmaß dieser hippokampalen Schädigung korreliert bei der LGI1-Enzephalitis mit dem Schweregrad persistierender Gedächtnisdefizite. Für Kinder mit NMDAR-Enzephalitis wurde gezeigt, dass MRT-Veränderungen zu Beginn der Erkrankung mit einem schlechteren Langzeitoutcome assoziiert sind [5]. Eine umfassende Übersicht über weitere bildgebende Veränderungen (MRT, PET) bei Autoimmunenzephalitiden findet sich bei Heine et al. [31].

### Liquor

Die Liquoranalyse ist ein weiterer zentraler Bestandteil der Diagnostik. Eine Pleozytose findet sich bei mehr als 50 % der Patienten mit Antikörpern gegen GABA_B_R, AMPAR, NMDAR oder DPPX, deutlich seltener jedoch – in absteigender Frequenz – bei Antikörpern gegen CASPR2, GABA_A_R, GlycinR, IgLON5, LGI1 und GAD [7]. Isolierte oligoklonale Banden im Liquor lassen sich bei mehr als 50 % der Patienten mit GABA_B_R-, NMDAR- und GAD-Antikörpern nachweisen; niedrigere Frequenzen finden sich – in absteigender Reihenfolge – bei Antikörpern gegen AMPAR, DPPX, CASPR2, GABA_A_R, GlycinR, IgLON5 und LGI1.

### EEG

EEG-Veränderungen sind häufig, aber selten spezifisch; zumeist findet sich eine fokale oder generalisierte Verlangsamung. Eine Ausnahme sind Beta-Delta-Komplexe („extreme delta brush“), die bei ca. 10 % der Patienten mit NMDAR-Enzephalitis nachweisbar sind und durch eine generalisierte Delta-Verlangsamung mit überlagerter Beta-Aktivität gekennzeichnet sind. Sie treten v. a. bei klinisch schwer betroffenen Patienten auf [52]. Wichtig ist, dass akutsymptomatische Anfälle im Akutstadium autoimmuner Enzephalitiden auch über mehrere Monate hinweg auftreten können und dennoch nur ein relativ geringes Risiko für die Entwicklung einer manifesten Epilepsie besteht, sodass eine dauerhafte antikonvulsive Therapie oft nicht erforderlich ist (im Gegensatz zur „autoimmunen Epilepsie“, bei der epileptische Anfälle ein fortbestehendes Kernsymptom der Erkrankung darstellen [25]).

### Antikörperdiagnostik

Bei allen Patienten mit Verdacht auf Autoimmunenzephalitis sollte eine Antikörperdiagnostik in Liquor und Serum erfolgen, auch wenn MRT, Liquor und EEG unauffällig sind (Abb. 4). Da die Antikörperdiagnostik für den sicheren Nachweis einer paraneoplastischen Ätiologie sowohl bei Hochrisikosyndromen für ein Karzinom (z. B. limbische Enzephalitis oder paraneoplastische Kleinhirndegeneration) als auch bei Syndromen mit mittlerem Krebsrisiko (z. B. Hirnstammenzephalitis oder Morvan-Syndrom) erforderlich ist [27], sollte eine möglichst breite Diagnostik inklusive der in Tab. 1 genannten Antikörper sowie zumindest GABA-B-R-Ak, GABA-A-R-Ak und AMPA-R-Ak umfassen.

Bei sonstigen Autoimmunenzephalitiden sollten immer NMDAR-, LGI1-, CASPR2-, AMPA-R- und GABA-R-Antikörper bestimmt werden, bei Zeichen einer Hyperexzitabilität (u. a. Myoklonien, Hyperekplexie, epileptische Anfälle, Delir) zusätzlich GlycinR-, DPPX- und GAD-Antikörper, bei Psychosen auch mGluR5-Antikörper (Tab. 2). Prinzipiell ist immer eine breite Panel-Diagnostik zu bevorzugen, die auch neue Antikörper wie IgLON5 enthält. Bei der NMDAR-Enzephalitis sind bei ca. 10–20 % der Patienten Antikörper nur im Liquor nachweisbar; hohe Titer weisen zudem auf eine ungünstigere Prognose hin. Einige Antikörper sind erst ab höheren Titern sicher krankheitsrelevant, z. B. CASPR2 (ab 1:320) und GAD (ab 2000 U/ml; [48]).

## Therapie

Eine Therapie sollte so früh wie möglich – gegebenenfalls auch bei noch ausstehendem Antikörpernachweis – eingeleitet werden. Als Erstlinientherapien etabliert sind eine Hochdosissteroidtherapie mit intravenös verabreichtem Methylprednisolon (in der Regel 1000 mg pro Tag über 5 Tage), therapeutische Aphereseverfahren (Plasmaaustausch, Immunadsorption; umtägig über 5 bis 10 Behandlungen) oder intravenöse Immunglobuline (2 g/kg Körpergewicht über 3 bis 5 Tage). Während Patienten mit einer LGI1-Enzephalitis unter Steroiden in der Regel frühzeitig eine deutliche Besserung faziobrachialer dystoner Anfälle erleben, reichen diese für eine langfristige Symptomkontrolle in der Regel bei keiner Enzephalitisform aus. Außer bei sehr milden Verläufen oder sehr ausgeprägter Besserung unter der Erstlinientherapie hat sich mittlerweile bei den meisten Enzephalitiden mit Oberflächenantikörpern eine Therapieerweiterung bzw. Rezidivprophylaxe etabliert, die in vielen Fällen mit Rituximab durchgeführt wird (1000 mg, im Abstand von 14 Tagen wiederholt, dann gegebenenfalls erneut in 6‑monatigen Abständen).

Traditionell wird als Therapieerweiterung auch Cyclophosphamid empfohlen, aufgrund der erheblichen Toxizität und möglichen Langzeitnebenwirkungen ist die Anwendung in vielen Zentren allerdings deutlich eingeschränkt worden. Neue Daten sprechen für eine Anwendung des Proteasominhibitors Bortezomib [6, 49], der derzeit auch in einer deutschen multizentrischen placebokontrollierten Investigator-initiierten Studie bei Patienten mit Autoimmunenzephalitis untersucht wird (GENERATE BOOST [60]). Kürzlich begannen weitere randomisierte klinische Therapiestudien, beispielweise zu Inebilizumab bei NMDAR-Enzephalitis [9] und zu Rozanolixizumab [10] und IVIG [16] in der Therapie der LGI1-Enzephalitis. In Einzelfällen kann zur langfristigen remissionserhaltenden Therapie auch eine steroidsparende immunsuppressive Therapie u. a. mit Methotrexat oder Azathioprin erwogen werden [12], belastbare Studiendaten hierzu liegen derzeit jedoch nicht vor.

Im Falle paraneoplastischer Autoimmunenzephalitiden mit Antikörpern gegen intrazelluläre Antigene ist die Antikörperelimination aufgrund der nicht direkt pathogenen Wirkung der Antikörper weniger wirkungsversprechend, eine zeitnahe Tumorsuche und -therapie steht klar im Vordergrund. Bereits entstandener neuronaler Schaden ist häufig irreversibel. Dennoch ist insbesondere in der Frühphase eine Immuntherapie zu versuchen, die aber vorrangig zytotoxische T‑Zellen eliminieren sollte (z. B. mittels Cyclophosphamid) und an spezialisierten Zentren die Anwendung erweiterter Chemotherapien oder Biologika (z. B. Alemtuzumab) einschließen kann.

Die Entfernung eines Ovarialteratoms bei Patientinnen mit einer NMDAR-Enzephalitis ist zügig erforderlich. Die noch vor wenigen Jahren in einigen Zentren praktizierte „blinde“ Ovarektomie bei schweren Verläufen ohne Teratomnachweis ist aus unserer Sicht nicht mehr zeitgemäß, da sich wahrscheinlich alle Teratome in einer Kombination aus Abdomen-MRT mit Kontrastmittel, PET-CT (vor allem bei mediastinalen Teratomen) und explorativer Laparoskopie detektieren lassen.

Darüber hinaus sollte eine akutsymptomatische Therapie entsprechend dem Beschwerdebild, z. B. mit Antiepileptika und Antipsychotika, nur in Zusammenhang mit einer Immuntherapie erwogen werden, da beispielweise Antikonvulsiva allein selten zu einer effektiven Kontrolle epileptischer Anfälle führen [58]. Während Antikonvulsiva nach erfolgreicher Immuntherapie einer NMDAR- oder GABA-A-R-Enzephalitis früh wieder abgesetzt werden können, bestehen bei Patienten nach limbischer Enzephalitis oft strukturelle Epilepsien fort. Auch nichtmedikamentöse Therapien wie Physiotherapie oder neuropsychologische Rehabilitation können hilfreich sein.

## Langzeitverlauf

Zum Langzeitverlauf von Autoimmunenzephalitiden existieren bisher nur wenige Daten. Bei der LGI1-Enzephalitis erholen sich nur 35 % der Patienten nach 2 Jahren vollständig [1]. Die Mehrzahl der Patienten leidet an persistierenden Gedächtnisdefiziten aufgrund einer strukturellen Schädigung und Atrophie des Hippokampus [22]. Ein verzögerter Therapiebeginn, fehlende Therapieansprache und Rezidive wurden als Prädiktoren für ein schlechtes Outcome identifiziert [1]. Bei der NMDAR-Enzephalitis erreicht die Mehrzahl der Patienten ein gutes bis sehr gutes neurologisches Outcome – gemessen anhand der (eigentlich für den Schlaganfall entwickelten) modified Rankin Scale (mRS; [59]). Im Gegensatz hierzu weisen jedoch bis zu 80 % der NMDAR-Enzephalitis-Patienten 2 Jahre nach Erkrankungsbeginn noch moderate bis hochgradige kognitive Defizite auf, die v. a. das Gedächtnis und die Exekutivfunktionen betreffen [30]. Prädiktoren für kognitive Defizite waren ebenfalls ein verzögerter Therapiebeginn sowie eine höhere Erkrankungsschwere. Etwa 5 Jahre nach der Akutphase (Median) war ein Drittel der Patienten genesen, während bei einem Drittel noch moderate und bei einem weiteren Drittel noch ausgeprägte kognitive Defizite zu finden waren. Die Genesung von neuropsychologischen Defiziten war zwar kurz nach der Akutphase am größten, jedoch konnte eine Verbesserung der kognitiven Funktionen noch mehrere Jahre nach Erkrankungsbeginn beobachtet werden, sodass eine fortgesetzte kognitive Rehabilitation dieser überwiegend noch sehr jungen Patienten angestrebt werden sollte.

## Fazit für die Praxis

- Für die meisten Autoimmunenzephalitiden sind die klinischen Manifestationen mittlerweile gut charakterisiert und international konsentierte diagnostische Kriterien etabliert.
- MRT, Liquor und EEG können unauffällig sein und Normalbefunde schließen eine Autoimmunenzephalitis daher nicht aus.
- Auch zur Therapie gibt es konsentierte Empfehlungen; randomisierte klinische Therapiestudien (z. B. zu Bortezomib, IVIG, Inebulizumab, Sartralizumab und Rozanolixizumab) laufen jedoch gerade erst an, in Deutschland vor allem koordiniert durch das Enzephalitis-Netzwerk GENERATE
- Eine frühzeitige und adäquate Therapie ist einer der wichtigsten Prädiktoren für ein gutes Outcome.
- Zum Langzeitverlauf gibt es bisher erst unzureichende Daten. Viele Patienten leiden jedoch an persistierenden kognitiven Defiziten, die bisher meist nur unzureichend rehabilitiert werden.
- Aufgrund der dynamischen Entwicklung des Autoimmunenzephalitis-Feldes ist auch in Zukunft mit der Charakterisierung zahlreicher neuer, pathogenetisch relevanter Antikörper, neuer klinischer Entitäten und neuer Assoziationen von Antikörpern und klinischen Korrelaten zu rechnen.

## References

[1] Arino H, Armangué T, Petit-Pedrol M (2016). Anti-LGI1-associated cognitive impairment Presentation and long-term outcome. Neurology.

[2] Ariño H, Gresa-Arribas N, Blanco Y (2014). Cerebellar ataxia and glutamic acid decarboxylase antibodies: immunologic profile and long-term effect of immunotherapy. JAMA Neurol.

[3] Armangue T, Spatola M, Vlagea A (2018). Frequency, symptoms, risk factors, and outcomes of autoimmune encephalitis after herpes simplex encephalitis: a prospective observational study and retrospective analysis. Lancet Neurol.

[4] Athauda D, Delamont RS, De Pablo-Fernandez E (2014). High grade Glioma mimicking voltage gated potassium channel complex associated antibody limbic encephalitis. Case Rep Neurol Med.

[5] Bartels F, Krohn S, Nikolaus M (2020). Clinical and magnetic resonance imaging outcome predictors in pediatric anti-N-methyl-D-Aspartate receptor encephalitis. Ann Neurol.

[6] Behrendt V, Krogias C, Reinacher-Schick A (2016). Bortezomib treatment for patients with anti-N-methyl-d-Aspartate receptor encephalitis. JAMA Neurol.

[7] Blinder T, Lewerenz J (2019). Cerebrospinal fluid findings in patients with autoimmune encephalitis—a systematic analysis. Front Neurol.

[8] Carvajal-González A, Leite MI, Waters P (2014). Glycine receptor antibodies in PERM and related syndromes: characteristics, clinical features and outcomes. Brain.

[9] ClinicalTrials.gov (2021) The ExTINGUISH Trial of Inebilizumab in NMDAR Encephalitis (ExTINGUISH). https://clinicaltrials.gov/ct2/show/NCT04372615. Zugegriffen: 09.12.2022

[10] ClinicalTrials.gov (2021) A study to test the efficacy, safety, and pharmacokinetics of rozanolixizumab in adult study participants with Leucine-Rich Glioma inactivated 1 autoimmune encephalitis. https://clinicaltrials.gov/ct2/show/NCT04875975. Zugegriffen: 09.12.2022

[11] van Coevorden-Hameete MH, de Graaff E, Titulaer MJ (2014). Molecular and cellular mechanisms underlying anti-neuronal antibody mediated disorders of the central nervous system. Autoimmun Rev.

[12] Dalmau J, Armangué T, Planagumà J (2019). An update on anti-NMDA receptor encephalitis for neurologists and psychiatrists: mechanisms and models. Lancet Neurol.

[13] Dalmau J, Geis C, Graus F (2017). Autoantibodies to synaptic receptors and neuronal cell surface proteins in autoimmune diseases of the central nervous system. Physiol Rev.

[14] Dalmau J, Lancaster E, Martinez-Hernandez E (2011). Clinical experience and laboratory investigations in patients with anti-NMDAR encephalitis. Lancet Neurol.

[15] Devine MF, Kothapalli N, Elkhooly M, Dubey D (2021). Paraneoplastic neurological syndromes: clinical presentations and management. Ther Adv Neurol Disord.

[16] Dubey D, Britton J, McKeon A (2020). Randomized placebo-controlled trial of intravenous immunoglobulin in autoimmune LGI1/CASPR2 epilepsy. Ann Neurol.

[17] Dubey D, Hinson SR, Jolliffe EA (2018). Autoimmune GFAP astrocytopathy: prospective evaluation of 90 patients in 1 year. J Neuroimmunol.

[18] Fang B, McKeon A, Hinson SR (2016). Autoimmune glial fibrillary acidic protein astrocytopathy. JAMA Neurol.

[19] Finke C, Kopp UA, Pajkert A (2016). Structural hippocampal damage following anti-N-methyl-D-Aspartate receptor encephalitis. Biol Psychiatry.

[20] Finke C, Kopp UA, Prüss H (2012). Cognitive deficits following anti-NMDA receptor encephalitis. J Neurol Neurosurg Psychiatry.

[21] Finke C, Kopp UA, Scheel M (2013). Functional and structural brain changes in anti-N-methyl-D-aspartate receptor encephalitis. Ann Neurol.

[22] Finke C, Prüss H, Heine J (2017). Evaluation of cognitive deficits and structural hippocampal damage in encephalitis with Leucine-Rich, glioma-inactivated 1 antibodies. JAMA Neurol.

[23] Franke C, Ferse C, Kreye J (2020). High frequency of cerebrospinal fluid autoantibodies in COVID-19 patients with neurological symptoms.

[24] Gaig C, Graus F, Compta Y (2017). Clinical manifestations of the anti-IgLON5 disease. Neurology.

[25] Geis C, Planagumà J, Carreño M (2019). Autoimmune seizures and epilepsy. J Clin Invest.

[26] Graus F, Titulaer MJ, Balu R (2016). A clinical approach to diagnosis of autoimmune encephalitis. Lancet Neurol.

[27] Graus F, Vogrig A, Muñiz-Castrillo S (2021). Updated diagnostic criteria for paraneoplastic neurologic syndromes. Neurol Neuroimmunol Neuroinflamm.

[28] Grüter T, Möllers FE, Tietz A (2022). Clinical, serological and genetic predictors of response to immunotherapy in anti-IgLON5 disease. Brain.

[29] Hara M, Ariño H, Petit-Pedrol M (2017). DPPX antibody-associated encephalitis. Neurology.

[30] Heine J, Kopp UA, Klag J (2021). Long-term cognitive outcome in anti-N-methyl-D-aspartate receptor encephalitis. Ann Neurol.

[31] Heine J, Prüss H, Bartsch T (2015). Imaging of autoimmune encephalitis—relevance for clinical practice and hippocampal function. Neuroscience.

[32] Heine J, Prüss H, Kopp UA (2018). Beyond the limbic system: disruption and functional compensation of large-scale brain networks in patients with anti-LGI1 encephalitis. J Neurol Neurosurg Psychiatry.

[33] Hoftberger R, van Sonderen A, Leypoldt F (2015). Encephalitis and AMPA receptor antibodies: novel findings in a case series of 22 patients. Neurology.

[34] Honorat JA, Komorowski L, Josephs KA (2017). IgLON5 antibody. Neurol Neuroimmunol Neuroinflamm.

[35] Hutchinson M, Waters P, McHugh J (2008). Progressive encephalomyelitis, rigidity, and myoclonus: a novel glycine receptor antibody. Neurology.

[36] Irani SR, Alexander S, Waters P (2010). Antibodies to Kv1 potassium channel-complex proteins leucine-rich, glioma inactivated 1 protein and contactin-associated protein-2 in limbic encephalitis, Morvan’s syndrome and acquired neuromyotonia. Brain.

[37] Kreye J, Reincke SM, Kornau H-C (2020). A therapeutic non-self-reactive SARS-coV-2 antibody protects from lung pathology in a COVID-19 hamster model. Cell.

[38] Lai M, Huijbers MG, Lancaster E (2010). Investigation of LGI1 as the antigen in limbic encephalitis previously attributed to potassium channels: a case series. Lancet Neurol.

[39] Lancaster E, Huijbers MGM, Bar V (2011). Investigations of caspr2, an autoantigen of encephalitis and neuromyotonia. Ann Neurol.

[40] Lancaster E, Lai M, Peng X (2010). Antibodies to the GABA(B) receptor in limbic encephalitis with seizures: case series and characterisation of the antigen. Lancet Neurol.

[41] López Chiriboga AS, Siegel JL, Tatum WO (2017). Striking basal ganglia imaging abnormalities in LGI1 ab faciobrachial dystonic seizures. Neurol Neuroimmunol Neuroinflamm.

[42] Muñoz-Lopetegi A, de Bruijn MAAM, Boukhrissi S (2020). Neurologic syndromes related to anti-GAD65. Neurol Neuroimmunol Neuroinflamm.

[43] Panariello A, Bassetti R, Radice A (2020). Anti-NMDA receptor encephalitis in a psychiatric Covid-19 patient: a case report. Brain Behav Immun.

[44] Peer M, Prüss H, Ben-Dayan I (2017). Functional connectivity of large-scale brain networks in patients with anti-NMDA receptor encephalitis: An observational study. Lancet Psychiatry.

[45] Prüss H (2017). Postviral autoimmune encephalitis. Curr Opin Neurol.

[46] Prüss H (2021). Autoantibodies in neurological disease. Nat Rev Immunol.

[47] Prüss H, Finke C, Höltje M, Hofmann J, Klingbeil C, Probst C, Borowski K, Ahnert-Hilger G, Harms L, Schwab JM, Ploner CJ, Komorowski L, Stoecker W, Dalmau J, Wandinger K‑P (2012). N‑methyl-D-aspartate receptor antibodies in herpes simplex encephalitis. Ann Neurol.

[48] Rössling R, Prüss H (2020). SOP: antibody-associated autoimmune encephalitis. Neurol Res Pract.

[49] Scheibe F, Prüss H, Mengel AM (2017). Bortezomib for treatment of therapy-refractory anti-NMDA receptor encephalitis. Neurology.

[50] Schubert J, Brämer D, Huttner HB (2019). Management and prognostic markers in patients with autoimmune encephalitis requiring ICU treatment. Neurol Neuroimmunol Neuroinflamm.

[51] von Schwanenflug N, Krohn S, Heine J (2020). State-dependent signatures of Anti-NMDA-Receptor Encephalitis: a dynamic functional connectivity study.

[52] van Sonderen A, Arends S, Tavy DLJ (2018). Predictive value of electroencephalography in anti-NMDA receptor encephalitis. J Neurol Neurosurg Psychiatry.

[53] van Sonderen A, Petit-Pedrol M, Dalmau J, Titulaer MJ (2017). The value of LGI1, Caspr2 and voltage-gated potassium channel antibodies in encephalitis. Nat Rev Neurol.

[54] van Sonderen A, Thijs RD, Coenders EC (2016). Anti-LGI1 encephalitis: clinical syndrome and long-term follow-up. Neurology.

[55] Spatola M, Petit-Pedrol M, Simabukuro MM (2017). Investigations in GABA A receptor antibody-associated encephalitis. Neurology.

[56] Spatola M, Sabater L, Planagumà J (2018). Encephalitis with mGluR5 antibodies. Neurology.

[57] Strippel C, Herrera-Rivero M, Wendorff M (2022). A genome-wide association study in autoimmune neurological syndromes with anti-GAD65 autoantibodies. Brain.

[58] Thompson J, Bi M, Murchison AG (2018). The importance of early immunotherapy in patients with faciobrachial dystonic seizures. Brain.

[59] Titulaer MJ, McCracken L, Gabilondo I (2013). Treatment and prognostic factors for long-term outcome in patients with anti-NMDA receptor encephalitis: an observational cohort study. Lancet Neurol.

[60] Wickel J, Chung H-Y, Platzer S (2020). Generate-Boost: study protocol for a prospective, multicenter, randomized controlled, double-blinded phase II trial to evaluate efficacy and safety of bortezomib in patients with severe autoimmune encephalitis. Trials.

